# Investigation of prognostic factors in patients with terminal pancreatic cancer: Focus on clinical symptoms, cachexia-related proteins, and body composition analysis at admission

**DOI:** 10.20407/fmj.2025-003

**Published:** 2025-08-06

**Authors:** Akihiro Ito, Masanobu Usui, Miyo Murai, Masanori Tsuzuki, Akihiko Futamura, Kazuki Imai, Yoshinori Itani

**Affiliations:** 1 Department of Surgery and Palliative Medicine, Fujita Health University, School of Medicine, Tsu, Mie, Japan; 2 Department of Medical Technology, Clinical Examination Division, Fujita Health University Nanakuri Memorial Hospital, Tsu, Mie, Japan

**Keywords:** Terminal pancreatic cancer, Prognostic factors, C-reactive protein, Extracellular water/total body water, Depression

## Abstract

**Objective:**

In palliative medicine, accurate prognosis of patients with terminal cancer is crucial. Serum albumin (Alb), transthyretin (TTR), and C-reactive protein (CRP) have been reported to be associated with prognosis. Pancreatic cancer is generally a rapidly progressing disease with an extremely poor prognosis. In the present study, we focused on identifying prognostic factors in patients with terminal pancreatic cancer.

**Methods:**

Overall, 756 patients with terminal cancer were admitted for palliative care and died during the 3-year period from April 2018; of these, 72 patients with pancreatic cancer (9.5%; excluding those who died within 7 days of admission) were included in the study. In addition to assessing clinical symptoms at admission, blood tests were performed to measure serum Alb (g/dL), TTR (mg/dL), CRP (mg/dL), and tumor markers. Body composition was analyzed using bioelectrical impedance analysis (BIA; InBody S10) to determine skeletal muscle mass index (SMI, kg/m^2^), extracellular water to total body water ratio (ECW/TBW), and phase angle (°). Prognostic factors were then evaluated.

**Results:**

The median age was 75 years (range: 33–91 years), with a male-to-female ratio of 37:35. The median survival time was 24 days (range: 7–99 days). After adjusting for age, sex, and performance status as potential confounders, multivariate analysis revealed that CRP (hazard ratio [HR]: 1.0998; 95% confidence interval [CI]: 1.0248–1.1781; *p*=0.0072), ECW/TBW (HR: 1.4391; 95% CI: 1.1429–1.8080; *p*=0.0018), and depressed mood (HR: 1.155; 95% CI: 1.036–1.281; *p*=0.0074) were significantly associated with prognosis. The cutoff values for 4-week survival (approximately 1-month survival) were CRP ≥2.0 mg/dL and ECW/TBW ≥0.430.

**Conclusion:**

Patients with terminal pancreatic cancer presented with advanced chronic inflammation at admission in addition to cachexia. Multivariate analysis revealed that serum CRP level and ECW/TBW ratio are independent prognostic factors, suggesting that prognosis in terminal pancreatic cancer is associated with the severity of chronic inflammation and edema. Among clinical symptoms, the degree of depression may also be related to prognosis, indicating that psychological distress should be considered in palliative care assessments.

## Introduction

In palliative care for patients with terminal cancer, accurately predicting prognosis is essential for providing high-quality medical care that aligns with the wishes of both patients and their families, while also maintaining quality of life until the end of life. Among terminal cancers, pancreatic cancer is one of the most refractory, with an extremely poor prognosis. In Japan, according to *Cancer Statistics 2024*, the annual number of cancer-related deaths is estimated at 385,797. By cancer type, the number of deaths due to pancreatic cancer has been increasing year by year, surpassing gastric cancer to become the third most common cause of cancer death.^1^ Furthermore, although the incidence of cachexia among patients with cancer varies depending on the type of cancer, it is most prevalent in pancreatic cancer.^2^ Patients with pancreatic cancer-related cachexia typically exhibit an increased systemic inflammatory response and decreased physical activity, both of which contribute to a significant decline in quality of life.^3^ For these reasons, predicting prognosis in terminal pancreatic cancer is particularly important. Previous studies have investigated prognostic factors relevant to palliative care. Murai et al. reported that interleukin (IL)-8 and clinical symptoms were associated with prognosis in advanced patients with cancer and cachexia, and that serum albumin (Alb) and transthyretin (TTR) levels were significantly lower in patients with a short prognosis compared to those with a longer prognosis.^4^ Miura et al. reported that, in the field of palliative care, the Glasgow Prognostic Score (GPS),^5^ which is defined by serum Alb and C-reactive protein (CRP) levels, serves as an important prognostic indicator in patients with cancer.^6^ In addition, Miura et al. found that serum TTR is not only a nutritional marker but also a significant prognostic factor in palliative settings.^7^ However, there are currently no studies that have investigated prognostic factors in patients with terminal cancer according to cancer type, such as pancreatic cancer.

Clinically, patients with terminal pancreatic cancer may present with ascites and pleural effusion due to carcinomatous peritonitis or pleurisy, liver dysfunction from multiple liver metastases, pericardial effusion, renal dysfunction, and edema resulting from malnutrition, anemia, or hypoalbuminemia. In this context, body composition analysis using bioelectrical impedance analysis (BIA) has recently attracted attention. The BIA method enables measurement of skeletal muscle mass, an indicator of sarcopenia, as well as the extracellular water to total body water ratio (ECW/TBW), an indicator of edema. In patients with terminal pancreatic cancer, these parameters are often abnormal. Although the utility of BIA as a sarcopenia index in patients with advanced pancreatic cancer undergoing anticancer treatment has been reported,^8^ there have been no studies examining its use for identifying prognostic factors in patients with terminal pancreatic cancer receiving palliative care. In the present study, we investigated the correlation between prognosis and cachexia-related proteins, tumor markers, BIA-based body composition metrics, and clinical symptoms in patients with terminal pancreatic cancer.

## Methods

Of 756 patients with terminal cancer who were admitted to Fujita Health University Nanakuri Memorial Hospital for palliative care during the 3-year period from April 2018 to March 2021 and died during hospitalization, 72 patients with pancreatic cancer (9.5%) were included in this study, excluding those who died within 7 days of admission. In this study, terminally ill patients with cancer were defined as those for whom multiple physicians determined—based on objective information such as diagnostic imaging and blood test results—that the disease was incurable, and who had had an estimated prognosis of approximately 3 months or less. This study was approved by the Ethics Review Committee of Fujita Health University (HM16-401).

Laboratory test items included general blood tests at admission, with serum transthyretin (TTR), albumin (Alb), and C-reactive protein (CRP) evaluated as cachexia-related markers, and carcinoembryonic antigen (CEA) and carbohydrate antigen (CA19-9) as tumor markers. Blood samples were collected in a fasting state before breakfast and analyzed from the same serum samples.

The reference values for serum TTR, Alb, CRP, CEA, and CA19-9 were 22–44 mg/dL, 4.0–5.0 g/dL, <0.7 mg/dL, <5.0 ng/mL, and <37 U/mL, respectively. Body composition was assessed using the InBody S100^®^ (InBody, Tokyo, Japan) within 2 days of admission. The skeletal muscle index (SMI: <7.0 kg/m^2^ in males and <5.7 kg/m^2^ in females) was used as an indicator of sarcopenia.^9^ The extracellular water to total body water ratio (ECW/TBW) served as an indicator of edema, and phase angle (PhA, °) as an index of cell membrane stability. SMI was calculated by dividing appendicular skeletal muscle mass by the square of the patient’s height.

**Figure 1:**
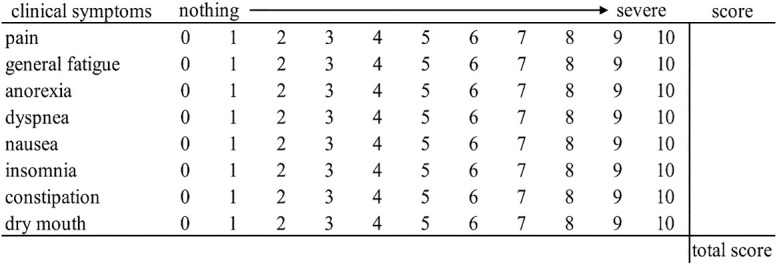
Overall assessment score

Clinical symptoms at admission were evaluated by scoring nine subjective symptoms—pain, general fatigue, anorexia, dyspnea, depression, nausea, insomnia, constipation, and dry mouth—on an 11-point scale from 0 to 10, following the national rating scale,^10^ which is commonly used to express pain. The total scores for these nine items were summed to create a comprehensive index (Figure 1). Symptom assessment was based on the Edmonton Symptom Assessment System (ESAS-r).^11^ Because clinical symptoms were based on patients’ self-assessment, 10 patients who could not be evaluated were excluded from the study.

Measurement results are presented as median values with interquartile ranges (IQRs) for both continuous and categorical variables. First, a multivariate analysis was performed to evaluate the relationship between each prognostic factor and survival, adjusting for age, sex, and performance status as potential confounding factors. Each prognostic factor was treated as an individual explanatory variable. For those factors found to be significantly associated with survival (*p*<0.05), multivariate analysis using the Cox proportional hazards model was performed to identify independent prognostic variables. For all such factors, Spearman’s correlation coefficient (r) was calculated to assess multicollinearity. If strong correlations were observed between explanatory variables (1.0>r>0.8 or –1.0<r<–0.8), those variables were evaluated separately in different models. For independent prognostic factors, patients were divided into two groups based on the median value, and survival curves were generated using the Kaplan–Meier method. Survival rates were compared using the log-rank test. Additionally, a survival cutoff of 4 weeks (approximately one month)—a significant milestone for patients with terminal cancer and their families—was used to calculate the area under the curve (AUC) and p-values. All statistical analyses were performed using JMP Pro version 14.0 (SAS Institute, Cary, NC, USA).

## Results

The median age was 75 years, with a male-to-female ratio of 37:35. The median survival from the date of admission was 24 days (interquartile range [IQR], 16.25–36). At admission, the median serum transthyretin (TTR), a cachexia-related protein, was 9.7 mg/dL (IQR, 6.6–13.2), and the median albumin (Alb) was 2.6 g/dL (IQR, 2.3–2.9), both lower than the reference values in all patients. Conversely, the median C-reactive protein (CRP) level was 3.6 mg/dL (IQR, 0.9–8.4), and 57 patients (79.2%) had values above the reference range. The median serum carcinoembryonic antigen (CEA) level was 21 ng/mL (IQR, 9.0–117), and the median carbohydrate antigen (CA19-9) level was elevated at 2,863 U/mL (IQR, 252–32,480) (Table 1).

The relationship between serum TTR, Alb, and CRP levels (cachexia-related proteins) and survival (in days), adjusted for age, sex, and performance status as potential confounders, is shown in Table 2-A. All three markers were considered prognostic factors (TTR: *p*<0.0001, Alb: *p*<0.0001, CRP: *p*=0.0004; Table 2-A).

Body composition analysis revealed that 66.7% of male patients had sarcopenia (SMI <7.0 kg/m^2^), with a low median SMI of 6.27 (IQR, 5.20–7.28). The median SMI for female patients was also low at 5.13 (IQR, 4.45–6.39), and 67.7% met the diagnostic criteria for sarcopenia (SMI <5.7 kg/m^2^). The median extracellular water to total body water ratio (ECW/TBW), an indicator of edema, was elevated at 0.427 (IQR, 0.417–0.437). Nearly all patients (96.9%) had ECW/TBW ≥0.40. The median phase angle (PhA) was low at 2.65° (IQR, 2.025–3.1) (Table 1). The relationships between survival (days) and SMI, ECW/TBW, and PhA—adjusted for age, sex, and performance status—are shown in Table 2-B. ECW/TBW and PhA were considered significant prognostic factors (ECW/TBW: *p*<0.0001, PhA: *p*=0.0004).

Next, multicollinearity was examined for serum TTR, Alb, CRP, ECW/TBW, and PhA—factors considered prognostic. A strong negative correlation was observed between ECW/TBW and PhA (r=–0.8011; Table 3).

Accordingly, multivariate analysis using the Cox proportional hazards model was performed separately: once with serum TTR, Alb, CRP, and ECW/TBW (Table 4-A), and again with serum TTR, Alb, CRP, and PhA (Table 4-B).

Multivariate analysis using the Cox proportional hazards model—with all prognostic factors as explanatory variables, adjusted for age, sex, and performance status—revealed that serum CRP (hazard ratio: 1.0998, 95% confidence interval [CI]: 1.0248–1.1781; *p*=0.0072) and ECW/TBW (hazard ratio: 1.4391, 95% CI: 1.1429–1.8080; *p*=0.0018) were independent prognostic factors.

Regarding clinical symptoms, anorexia had the highest median score of 5 points (IQR, 2–7.25), followed by fatigue (2.5 points; IQR, 0–5) and pain (2 points; IQR, 0–4). The median total symptom score was 19.5 (IQR, 11–32) (Table 1).

The relationships between the nine clinical symptoms, the total score, and survival (in days)—adjusted for age, sex, and performance status—are shown in Table 5. Among clinical symptoms, depression was found to be significantly associated with prognosis (*p*=0.0103; Table 6).

**Figure 2:**
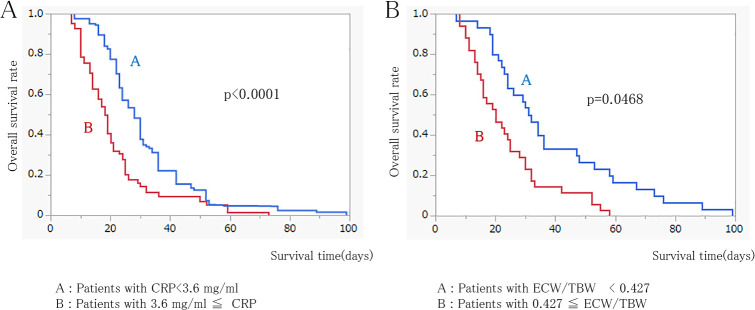
A: Comparison of survival rates by median CRP level. B: Comparison of survival rates by median ECW/TBW level.

**Figure 3:**
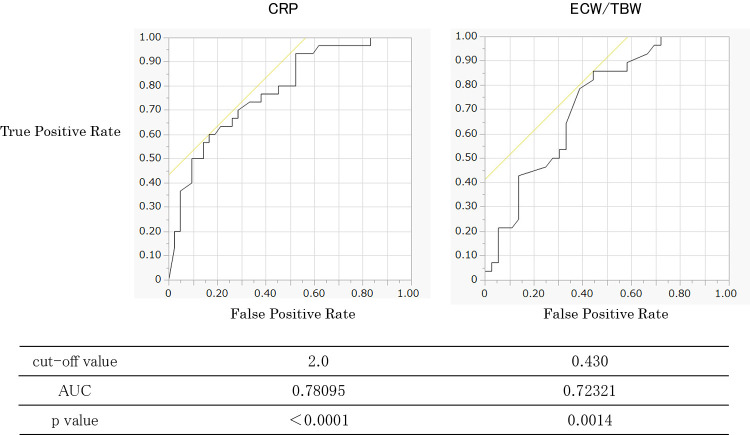
Cut-off value setting using ROC curve (4-week survival)

Furthermore, when all patients were divided into two groups based on the median CRP level (3.6 mg/dL), those with CRP ≥3.6 had significantly shorter median survival than those with CRP <3.6 (18.0 vs. 28.0 days; *p*<0.0001). Similarly, dividing patients by the median ECW/TBW (0.427) revealed that patients with ECW/TBW ≥0.427 had significantly shorter survival than those with ECW/TBW <0.427 (21.0 vs. 31.5 days; *p*=0.0468) (Figure 2-A, B). A regression equation predicting survival (in days) was derived using analysis of covariance on continuous data: Survival days=253.44–1.64×CRP (mg/dL)–5.02×ECW/TBW (%). Lastly, for a survival threshold of 4 weeks (approximately one month)—a key milestone for patients with terminal cancer and their families—the cutoff values and AUCs were as follows: CRP: cutoff=2.0 mg/dL, AUC=0.78095; ECW/TBW: cutoff=0.430, AUC=0.72321 (Figure 3).

## Discussion

Cachexia in patients with terminal cancer presents as significant loss of skeletal muscle and adipose tissue.^12,13^ Pathophysiologically, cachexia is characterized by a negative protein–energy balance resulting from decreased oral intake and metabolic abnormalities.^14^ In particular, pancreatic cancer often leads to reduced oral intake, which readily causes cachexia and deteriorates the patient’s general condition.^15^ Therefore, we investigated the relationship between serum TTR, Alb, and CRP levels—cachexia-related proteins—and survival (in days). The results revealed that serum CRP level is an independent prognostic factor in patients with terminal pancreatic cancer.

Shrotriya et al. reported in a systematic review that CRP is an important biomarker for prognosis in patients with recurrent solid tumors.^16^ In other words, CRP is produced as a biological response to cancer tissue invasion, metastasis, and the destruction of surrounding tissue, which triggers inflammatory reactions. Elevated serum CRP levels indicate higher degrees of tissue destruction and inflammation, which, in turn, suggest a worse prognosis. Furthermore, CRP is correlated with blood interleukin-6 (IL-6) levels, a marker that reflects tumor growth activity.^17^ Thus, CRP levels in patients with cancer may reflect both tumor burden and grade. Based on these factors, CRP was considered an independent prognostic factor even in patients with terminal pancreatic cancer. CRP is also an acute-phase protein and its levels increase sharply during acute bacterial infections such as pneumonia. In the present study, a detailed review of the 72 patients with terminal pancreatic cancer revealed that none had acute bacterial infections at the time of admission. Therefore, CRP levels in this study are believed to reflect chronic inflammation associated with cancer, which further supports its role as an independent prognostic factor. Nonetheless, acute infection should be ruled out when evaluating CRP, and this remains a consideration for future research.

Second, the BIA (bioelectrical impedance analysis) method has recently gained recognition as a diagnostic criterion for sarcopenia, and its utility in body composition analysis has been widely reported. BIA is a quick, non-invasive, and reproducible technique that utilizes the conductive properties of intracellular and extracellular fluids by applying electrical currents of various frequencies. Given that different tissues exhibit distinct electrical conductivities; BIA allows site-specific analysis of body composition. It is also effective for tracking dynamic physiological changes in patients with cancer. Previous studies have reported the usefulness of BIA as an index of sarcopenia in patients with advanced pancreatic cancer undergoing anticancer therapy.^8^ In the present study, we hypothesized that patients with terminal pancreatic cancer would exhibit advanced cachexia and worsening sarcopenia. However, we found no significant correlation between SMI (skeletal muscle index)—a key measure of sarcopenia—and prognosis. This may be due to increased fluid retention in muscle tissue, which could have artificially inflated muscle mass readings, thus affecting SMI accuracy in terminally ill patients with edema.

PhA (phase angle) is the phase difference between the resistance generated when current flows through body fluids and the reactance generated as it passes through cell membranes. In other words, the phase angle reflects the structural stability of cell membranes and overall cellular health. Although no standardized reference value has been established for phase angle, lower values are generally associated with poorer cell function. However, phase angle is also proportional to body size and tends to decrease with age. In the present study of patients with terminal pancreatic cancer, phase angle was not identified as an independent prognostic factor. This may be attributed to the decline in cellular function as cancer progresses, which could lead to a decrease in phase angle; however, other contributing factors may also exist, and thus PhA was not considered an independent prognostic factor.

The ECW/TBW ratio is an excellent indicator of the degree of edema in the body. In the general healthy population, the ECW/TBW ratio is approximately 0.38, and values greater than 0.40 are considered indicative of overhydration.^18^ In the presence of edematous conditions, this value tends to be higher due primarily to increased ECW. Furthermore, in cases of deteriorating nutritional status—such as in aging or sarcopenia—ICW (intracellular water) decreases, also resulting in a higher ECW/TBW ratio. Although ECW/TBW serves as an indicator of edema, it is also widely used as a measure of nutritional status and disease severity. Zheng et al. reported the utility of the BIA method in patients with advanced cancer, particularly noting that ECW/TBW ≥0.40 was a risk factor for poor prognosis.^19^ However, to our knowledge, no prior studies have examined the usefulness of body composition analysis as a disease-specific prognostic factor in patients with terminal cancer, especially those with pancreatic cancer. Therefore, we investigated the relationship between body composition and prognosis in the present study. Our results revealed that the ECW/TBW ratio is an independent prognostic factor in patients with terminal pancreatic cancer. This finding is likely due to the progression of cancer worsening nutritional status, leading to a reduction in ICW and an increase in ECW as a result of hypoalbuminemia-related edema. Additionally, fluid retention caused by ascites and pleural effusion related to cancer peritonitis and pleurisy, liver dysfunction due to multiple liver metastases, cardiac effusion, and renal dysfunction-related edema contribute to increased ECW. A high systemic inflammatory response, as part of the pathophysiology of cachexia, may also play a role by increasing vascular endothelial fragility and permeability, leading to further ECW accumulation. Therefore, the ECW/TBW ratio was considered an independent prognostic factor for patients with terminal pancreatic cancer. In the future, based on body composition measurements—which can be easily performed at the time of admission—we aim to attempt therapeutic interventions, such as administering tailored infusion therapies and diuretics in cases of observed fluid retention, and to evaluate the potential benefits and drawbacks of such approaches.

Finally, the clinical symptoms and prognosis of patients with terminal pancreatic cancer were examined. Murai et al. reported that clinical symptoms such as general fatigue, anorexia, dyspnea, and depression were prognostic factors in patients with advanced cancer and cachexia.^4^ Amano et al. also found that symptoms like general fatigue and anorexia worsened alongside elevated C-reactive protein (CRP) levels in patients with cancer receiving palliative care.^20^ Based on these findings, we investigated the relationship between clinical symptoms at admission and prognosis in patients with terminal pancreatic cancer.

In our study, depression appeared to be associated with prognosis based on multivariate analysis. However, this assessment was not objective; rather, it relied on a subjective 10-point pain score reported by terminally ill patients with pancreatic cancer, raising concerns about the ambiguity of the data and suggesting only a possible correlation with prognosis. Murai et al. found that, in patients with advanced cancer and cachexia, clinical symptoms—including depression, general fatigue, anorexia, dyspnea, nausea, and dry mouth—were significantly more severe in the short prognosis group than in the long prognosis group.^4^ Regarding depression, several factors may have contributed to its association with prognosis: (1) difficulty in maintaining supportive relationships with family members and healthcare providers,^21^ (2) loss of hope, which often results in passive symptom management even during palliative care, and (3) a sense of helplessness. These factors suggest a link between depression and prognosis. In the future, based on clinical symptom assessments (particularly depression) at the time of admission, we aim to evaluate the effectiveness and feasibility of early multidisciplinary interventions, team conferences, and, where appropriate, psychiatric intervention by specialists. In the present study, we identified two limitations: (1) we did not examine in detail whether the cause of death was due to a chronic cancer-related condition or an acute condition, such as gastrointestinal perforation, hemorrhage, or pneumonia; and (2) we did not assess whether nutritional management strategies—such as oral nutrition only, intravenous nutrition, or total parenteral nutrition—were implemented before and after admission to our hospital. These factors may have influenced patient survival (measured in days), and thus should be addressed in future research.

## Conclusion

In patients with terminal pancreatic cancer, the nutritional and cachexia indices—serum transthyretin (TTR) and albumin (Alb) levels—were below the reference values in all cases. Conversely, many patients exhibited chronic inflammatory conditions, with CRP levels exceeding the reference range. Multivariate analysis revealed that serum CRP levels and the ECW/TBW ratio from body composition analysis were independent prognostic factors. These findings suggest that the prognosis of patients with terminal pancreatic cancer may be more closely related to the degree of chronic inflammation and edema than to nutritional status or sarcopenia. Additionally, the severity of depression may also be associated with prognosis, indicating that psychological distress should be taken into consideration.

## Figures and Tables

**Table 1:** Patient characteristics

Factor	n=72
Age (years, median)	73 (33–96)
Sex, n (%)	72
male	37 (51.4)
female	35 (48.6)
Body mass index (kg/m^2^: IQR)	18.1 (16.3–20.9)
ECOG PS, n (%)	
1	0 (0)
2	18 (25.0)
3	40 (55.6)
4	14 (19.4)
Laboratory data	
Transthyretin (mg/dL: IQR)	9.7 (6.6–13.2)
Albumin (g/dL: IQR)	2.6 (2.3–2.9)
C-reactive protein (mg/dL: IQR)	3.6 (0.9–8.4)
Carcinoembryonic antigen (ng/mL: IQR)	21 (9.0–117)
Carbohydrate antigen 19-9 (U/mL: IQR)	2863 (252–32480)
Clinical symptoms	
Pain (IQR)	2 (0–4)
General fatigue (IQR)	2.5 (0–5)
Anorexia (IQR)	5 (2–7.25)
Dyspnea (IQR)	0 (0–2)
Depression (IQR)	2 (0–4.25)
Nausea (IQR)	0 (0–2)
Insomnia (IQR)	0 (0–4.25)
Constipation (IQR)	0 (0–2.25)
Drymouth (IQR)	0 (0–5.25)
Overall assessment (IQR)	19.5 (11–32.5)
Body composition analysis	
Skeletal muscle mass index (kg/m^2^: IQR)	
Male	6.27 (5.20–7.28)
Female	5.13 (4.45–6.39)
ECW/TBW (: IQR)	0.427 (0.417–0.437)
Phase angle (°: IQR)	2.65 (2.025–3.1)
Hospital stay (days: IQR)	24 (16.25–36)

**Table 2-A:** Significant differences between laboratory data and prognosis at admission

Laboratory data	Multivariable analysis: Prognosis (days)		
	Hazard ratio	95%CI	p value
Transthyretin	0.8898	0.8376–0.9420	<0.0001
Albumin	0.3189	0.1787–0.5692	<0.0001
C-reactive protein	1.1325	1.0588–1.2113	0.0004
Carcinoembryonic antigen	1.0000	0.9999–1.0001	0.8554
Carbohydrate antigen 19-9	1.0000	1.0000–1.0000	0.4200

※: adjustment for age, sex and performance status (PS) CI: confidence interval

**Table 2-B:** Significant differences between body composition analysis and prognosis at admission

	Multivariable analysis: Prognosis (days)		
	Hazard ratio	95%CI	p value
Skeletal muscle mass index			
Male	1.4587	1.0924–1.9442	0.1565
Female	1.2130	0.7947–1.8283	0.0565
ECW/TBW×100 (%)	1.6072	1.3232–1.9552	<0.0001
Phase angle	0.5224	0.3508–0.7555	0.0004

※: adjustment for age, sex and performance status (PS) CI: confidence interval

**Table 3:** Correlation between covariates—Spearman rank correlation coefficient

	TTR	Alb	CRP	ECW/TBW	Phase angle
TTR	1	0.5367	–0.4833	–0.4616	0.4372
Alb	0.5367	1	–0.4316	–0.4708	0.5536
CRP	–0.4833	–0.4316	1	0.1991	–0.1201
ECW/TBW	–0.4616	–0.4708	0.1991	1	–0.8011
Phase angle	0.4372	0.5536	–0.1201	–0.8011	1

**Table 4-A:** Multivariable Cox regression analysis for mortality in patients with terminal pancreatic cancer

	Multivariable analysis: Prognosis (days)		
	Hazard ratio	95%CI	p value
Age	0.9919	0.9677–1.0199	0.5436
Sex			0.4913
male/female	1.2103	0.7028–2.0842	0.4913
female/male	0.8262	0.4798–1.4228	0.4913
ECOG PS			0.1310
3/2	1.6785	0.8571–3.2871	0.1309
4/2	2.3677	1.0056–5.6177	0.0485
4/3	1.4160	0.6968–2.8766	0.3363
Transthyretin	0.9528	0.8806–1.0300	0.2244
Albumin	0.8589	0.4137–1.6894	0.6710
C–reactive protein	1.0998	1.0248–1.1781	0.0072
ECW/TBW×100 (%)	1.4391	1.1429–1.8080	0.0018

※: adjustment for age, sex and performance status CI: confidence interval; ECOG PS: Eastern Cooperative Oncology Group performance status

**Table 4-B:** Multivariable Cox regression analysis for mortality in patients with terminal pancreatic cancer

	Multivariable analysis: Prognosis (days)		
	Hazard ratio	95%CI	p value
Age	0.9948	0.9717–1.0212	0.6815
Sex			0.5936
male/female	1.1629	0.6679–2.0248	0.5936
female/male	0.8599	0.4939–1.4971	0.5936
ECOG PS			0.3745
3/2	1.4854	0.7533–2.9287	0.2533
4/2	1.7739	0.7691–4.0917	0.1789
4/3	1.1942	0.6067–2.3506	0.6074
Transthyretin	0.9549	0.8734–1.0228	0.1621
Albumin	0.8795	0.3983–1.8706	0.7442
C-reactive protein	1.1731	1.0341–1.2064	0.0046
Phase angle	0.6346	0.3728–1.0316	0.0790

※: adjustment for age, sex and performance status CI: confidence interval; ECOG PS: Eastern Cooperative Oncology Group performance status

**Table 5:** Significant differences between clinical symptoms and prognosis at admission

	Multivariable analysis: Prognosis (days)		
	Hazard ratio	95%CI	p value
Pain	1.0516	0.9108–1.2142	0.1704
General fatigue	1.1352	1.0410–1.2380	0.0747
Anorexia	1.1102	1.0218–1.2074	0.1070
Dyspnea	1.0973	0.9642–1.2773	0.2665
Depression	1.1552	1.0359–1.2809	0.0103
Nausea	1.0361	0.9187–1.1486	0.3610
Insomnia	0.9460	0.8589–1.0420	0.5165
Constipation	0.9250	0.8173–1.0305	0.1805
Drymouth	1.0360	0.9470–1.1267	0.4869
Overall assessment	1.0174	0.9963–1.0390	0.1605

**Table 6:** Multivariable Cox regression analysis for mortality in patients with terminal pancreatic cancer

	Multivariable analysis: Prognosis (days)		
	Hazard ratio	95%CI	p value
Age	0.9886	0.9665–1.0132	0.3505
Sex			0.1156
male/female	1.5370	0.8998–2.6254	0.1156
female/male	0.6506	0.3809–1.1114	0.1156
ECOG PS			0.0087
3/2	1.3682	0.7254–2.5805	0.3328
4/2	4.4378	1.8312–10.7545	0.0010
4/3	3.2435	1.4619–7.1962	0.0038
Depression	1.1552	1.0359–1.2809	0.0103

CI: confidence interval; ECOG PS: Eastern Cooperative Oncology Group performance status

## Supplementary Material

PDF-Japanese
