# Supplementary material for: Investigation of prognostic factors in patients with terminal pancreatic cancer: Focus on clinical symptoms, cachexia-related proteins, and body composition analysis at admission
Source: Fujita Med J. 2025 Aug 6;11(4):170–7. doi: 10.20407/fmj.2025-003 (PMC12576404; doi:10.20407/fmj.2025-003)
Supplement: Supplementary file 1 — PDF-Japanese [file fmj-11-170-s001.pdf]

Original article

Title

終末期膵がん患者の予後規定因子に関する検討、特に入院時臨床症状、悪液質関連蛋白、体組成分析を中心に

Prognostic factors in pancreatic terminal cancer patients, Especially focusing on clinical symptoms, cachexia-related proteins and body composition analysis on admission

Running Title
終末期膵がん患者の予後規定因子に関する検討
Prognostic factors in pancreatic terminal cancer patients

Akihiro Ito, MD, PhD[1]*, Masanobu Usui, MD, PhD[1]*, Miyo Murai, MD, PhD[1], Masanori Tsuzuki, MD, PhD[1], Akihiko Futamura, PhD[1], Kazuki Imai[1], Yoshinori Itani[2],

* These two authors contributed equally to this article
[1] Department of Surgery and Palliative Medicine, Fujita Health University, School of Medicine, Tsu, Mie, Japan
[2] Department of Medical Technology, Clinical Examination Division, Fujita Health University Nanakuri Memorial Hospital, Tsu, Mie, Japan

Corresponding Author： Akihiro Ito, MD, PhD
Department of Surgery and Palliative Medicine, Fujita Health University, School of Medicine, 424-1, Oodori-cho, Tsu, Mie 514-1295, Japan
TEL：059-252-1555
E-mail: itoaki@fujita-hu.ac.jp

要旨
【目的】緩和医療において、終末期がん患者の予後を予測することは、極めて重要である。これまで、血清アルブミン（Alb）、トランスサイレチン（TTR）、C-reactive protein(CRP)などが予後と関連することが報告されている。膵がんは、一般的に進行が速く極めて予後不良な疾患である。そこで、今回終末期膵がん患者の予後規定因子を中心に検討した。
【対象と方法】2018 年 4 月からの 3 年間に緩和医療目的で入院、死亡退院した終末期がん患者 756 例中、膵がん 72 例（9.5%：入院後 7 日未満死亡を除く）を対象とした。入院時の臨床症状に加え、血液検査として血清 Alb（g/dL）、TTR（mg/dL）、CRP（g/dL）値、腫瘍マーカーを測定、さらに、BIA 法による体組成分析（In Body S10）を実施し、SMI（skeletal muscle mass index：kg/m^2）、細胞外水分比（ECW/TBW）、Phase angle（°）を測定、予後規定因子を検討した。
【結果】年齢の中央値は、75(33-91）歳で、男女比は 37:35、予後日数の中央値は 24（7-99）日であった。年齢、性別、Performance status を潜在的な交絡因子として調節した後、血液、体組成データにおいては、CRP(hazard ratio 1.0998, 95% confidence interval 1.0248-1.1781; p = 0.0072)、ECW/TBW(hazard ratio 1.4391, 95% confidence interval 1.1429–1.8080; p = 0.0018)、臨床症状においては、気分の落ち込み (hazard ratio 1.155, 95% confidence interval 1.036–1.281; p = 0.0074)、が予後と関係する可能性がある考えられた。4 週間（約 1 カ月）生存におけるカットオフ値は CRP2.0mg/dL、ECW/ TBW0.430 であった。
【結語】入院時評価において、終末期膵がん患者は、悪液質に加え、高度慢性炎症状態であった。多変量解析より、血清 CRP 値や体組成分析での ECW/TBW が独立した予後規定因子であることより、終末期膵がん患者の予後は、慢性炎症、浮腫の程度に関与すると考えられた。臨床症状では、Depression の程度が予後と関係する可能性があることより、精神的苦痛も考慮しなければいけないと考えられた。

Key words： Pancreatic terminal cancer, Prognostic factor, C-reactive protein , Extracellular water / total body water, Depression

【序論：Introduction】終末期がん患者を対象とする緩和医療においては、患者やその家族の意向を反映した質の高い医療を提供し、最期の時が訪れるまで生活の質を維持するために、予後を予測することは、きわめて重要である。その中でも、膵がんは、難治性がんの 1 つであり、極めて予後が不良である。本邦において、がんの統計 2024 によれば、年間死亡者数は 385,797 人、がん種別では胃がんを抜いて 3 位となり、膵がんで死亡する患者数は年々増加している[1]。さらに、がん患者が陥る悪液質の発生頻度はがん種により異なるが、膵がんは上位を占め[2]、膵がんによる悪液質患者は全身性炎症反応の亢進と身体活動性の低下を特徴とするため、容易に QOL が低下する[3]。このためにも、終末期膵がん患者の予後を予測することは重要である。これまでに、緩和医療に関する予後関連因子としては、Murai らは、悪液質を伴う進行がん患者に対して、Interleukin-8 や臨床症状が予後規定因子であり、予後が短い群は、長い群に比較して、血清 Albumin (Alb)、Transthyretin(TTR)値は、有意に低値であり、C-reactive protein(CRP)値は、有意に高値であると報告している[4]。Miura らは、緩和ケア領域におけるがん患者に対して、血清 Alb、CRP 値で規定される Glasgow Prognostic Score (GPS)[5]が、予後予測因子として重要であると報告している[6]。さらに、Miura らは、血清 TTR 値も、緩和ケア領域における栄養指標であり、予後予測因子として重要であると報告している[7]。しかし、膵がんなど、がん種別に終末期がんの予後予測因子を検討した報告はない。

一方、終末期膵がん患者は、癌性腹膜炎や胸膜炎により腹水・胸水貯留、多発性肝転移による肝機能障害、心のう液貯留、腎機能障害、あるいは栄養障害や貧血、低アルブミン血症でも浮腫を併発することを臨床的に経験する。このようななかで、近年、Bioelectrical Impedance Analysis (BIA）による体組成分析が着目されている。BIA 法を用いることにより、サルコペニアの指標である筋肉量、浮腫の指標である細胞外水分比などの測定が可能である。終末期膵がん患者において、これらの測定値は異常となることが予想される。抗がん治療を行っている進行膵がん患者のサルコペニア指標としての BIA の有用性の報告[8]はみられるが、緩和医療が必要な終末期膵がん患者の予後予測因子に関し、BIA を検討した報告はない。そこで、今回、終末期膵がん患者における悪液質に関する蛋白や腫瘍マーカー、BIA による体組成分析や臨床症状と予後と

の相関関係ついて検討した。

【方法：Methods】

2018 年 4 月から 2021 年 3 月までの 3 年間に緩和医療目的で藤田医科大学　七栗記念病院に入院し、死亡退院した終末期がん患者 756 例中、膵がん 72 例（9.5％：入院後 7 日未満死亡を除く）を対象とした。今回、終末期がん患者は、複数の医師が客観的な情報（画像診断、血液検査など）を基に、治療により病気の回復ができない、推定される予後が約 3 カ月以内と判断される症例と定義した。本研究は、藤田医科大学医学研究倫理審査委員会（HM16-401）にて、承認されている。

検査項目は、入院時血液一般検査として、悪液質関連指標である、血清 TTR、Alb、CRP を、腫瘍マーカーである、carcinoembryonic antigen(CEA)、carbohydrate antigen (CA)19-9 を測定した。採血は、朝食前 空腹時に行い、同一血清を用いて分析した。

血清 TTR 濃度の基準値は、22~44mg/dL、Alb 濃度の基準値は、4.0~5.0g/dL、CRP 濃度の基準値は、~0.7mg/dL、CEA 濃度の基準値は、~5.0ng/mL、CA19-9 濃度の基準値は、~37U/mL であった。体組成は、入院後 2 日以内に、InBody S100® (InBody, Tokyo, Japan)を用いて評価した。サルコペニアの指標 [9] として、skeletal muscle index (SMI：男性<7.0、女性<5.7kg/m^2)を、浮腫の指標として、細胞外水分比（extracellular water：ECW/ total body water ：TBW）、細胞膜の安定性の指標として、Phase angle（phA：°）を評価した。SMI は、四肢の骨格筋量を身長の 2 乗で割って計算した。

入院時の臨床症状は、疼痛の表現方法である numerical rating scale[10] に倣って、以下の 9 項目の自覚的所見を 0–10 の 11 段階にスコアー化し、評価した：pain, general fatigue, anorexia, dyspnea, depression, nausea, insomnia, constipation, and dry mouth. これら 9 項目のスコアーを合計し、総合的な指標を作成し、評価した(Figure 1) 。臨床症状の評価は、Edmonton Symptom Assessment System (ESAS-r) [11] を参考に作成した。 本研究では、臨床症状は患者の主観的評価によって決定された。このため、患者が評価できない 10 例は対象から除外した。

測定結果は、連続変数、カテゴリー変数ともに、四分位範囲（IQRs）の中央値で表した。最初に、生存期間との関係を年齢、性別、Performance status を潜在的な交絡因子として調節し、それぞれの測

定結果（予後因子）を一つずつ説明変数として、多変量解析を行った。さらに、生存期間と有意な関係あり（P<0.05）と判断された予後因子に関して、すべての因子を説明変数として、Cox 比例ハザードモデルによる多変量解析を行い、独立した予後規定因子を評価した。その際、生存期間と有意な関係あり（p<0.05）と判断されたすべての因子に関しては、多重共線性を考慮し、Spearman の相関係数(r)を用いてそれぞれの相関係数を評価した。説明変数の間に、強い相関を持つ組み合わせ（1.0＞ r ＞0.8、-1.0＜ r <-0.8：多重共線性）が存在する場合は、同一検定とせず、別々に評価した。独立した予後規定因子に関しては、中央値にて 2 群に分け、Kaplan–Meier curves を用いて生存曲線を示し、生存率を log-rank test にて評価した。さらに、終末期がん患者、家族にとっての一つの節目の時期と考えられる 4 週間（約 1 カ月）生存におけるカットオフ値を設定し、AUC、p 値を検討した。すべての統計およびデータ分析は、JMP　Pro バージョン 14.0 ソフトウェア (SAS、米国ノースカロライナ州ケアリー) を使用して実行した。

【結果：Results】

平均年齢中央値は、75 歳で、男女比は 37:35 であった。入院日からの予後日数の中央値は 24（IQR、16.25-36）日であった。入院時、悪液質関連蛋白である血清 TTR の中央値は、9.7（IQR、6.6-13.2） Alb の中央値は、2.6（IQR、2.3-2.9）と全例基準値未満の低値であった。逆に、CRP の中央値は、3.6（IQR、0.9-8.4）で 57 例（79.2%）が基準値を超える高値であった。腫瘍マーカーである血清 CEA の中央値は、21（IQR、9.0-117)、血清 CA19-9 の中央値は、2863（IQR、252-32480）と高値であった（Table1)。

年齢、性別、Performance status を潜在的な交絡因子として調節した後、悪液質関連蛋白である、血清 TTR、Alb、CRP 値と予後日数の関係を Table2-A に示す。血清 TTR、Alb、CRP 値は、予後因子と考えられた（TTR： p<0.0001、Alb：p<0.0001、CRP： p=0.0004：Table 2-A)。

体組成分析の結果からは、男性の SMI の中央値は、6.27（IQR、5.20-7.28）と低値で 66.7%がサルコペニア（<7.0kg/m^2）と診断された。女性の SMI の中央値も、5.13（IQR、4.45-6.39）と低値で、67.7%がサルコペニア（<5.7kg/m^2）と診断された。浮腫の指標とし

ての、ECW/ TBW の中央値は、0.427（IQR、0.417-0.437）と高値であった。浮腫の指標である ECW/ TBW≧0.40 の症例は、96.9%とほぼすべての症例が該当した。PhA の中央値は、2.65（IQR、2.025-3.1）と低値であった（Table1)。年齢、性別、Performance status を潜在的な交絡因子として調節した後、SMI 、ECW/ TBW、PhA と予後日数の関係を Table2-B に示す。ECW/ TBW、PhA 値は、予後因子と考えられた（ECW/ TBW： p<0.0001、PhA：p＝0.0004)。
次に、予後因子と考えられた血清 TTR、Alb、CRP、ECW/ TBW、PhA 値につき、多重共線性を検討した。その結果、ECW/ TBW と PhA 値は、r=-0.8011 と強い負の相関関係が認められた（Table 3)。このため、血清 TTR、Alb、CRP 値、ECW/ TBW 値（Table 4-A）、血清 TTR、Alb、CRP 値、PhA 値（Table 4-B）に分け、Cox 比例ハザードモデルを用いた多変量解析を行った。
年齢、性別、Performance status を調整し、すべての予後因子を説明変数として、Cox 比例ハザードモデルによる多変量解析を行った結果、血清 CRP (hazard ratio 1.0998, 95% confidence interval 1.0248-1.1781; p= 0.0072)、ECW/TBW(hazard ratio 1.4391, 95% confidence interval 1.1429–1.8080; p = 0.0018)が独立した予後因子であると考えられた。

臨床症状では、食欲不振が、中央値 5 点（IQR、2-7.25）と最も高値で、次に倦怠感が中央値 2.5 点（IQR、0-5)、痛み中央値 2 点（IQR、0-4）の順であった。総合得点は、中央値 19.5 点（IQR、11-32）であった（Table 1)。

同様に、年齢、性別、Performance status を潜在的な交絡因子として調節した後、9 つの臨床症状、総合得点と予後日数の関係を Table5 に示す。臨床症状において Depression は、p=0.0103 と予後と関係する可能性が示唆された（Table 6)。

さらに、全患者を血清 CRP 値中央値（3.6mg/dL）にて 2 群に分け、予後日数中央値を検討すると、CRP≧3.6 は<3.6 と比較し、有意に短かった（18.0 vs 28.0, p<0.0001)。ECW/ TBW も中央値（0.427）にて 2 群に分け、予後日数中央値を検討すると、ECW/ TBW≧0.427 は <0.427 と比較し、有意に短かった（21.0 vs 31.5, p=0.0468)（Figure2-A,B)。連続データにおいて共分散分析を施行した結果、予後予測日数=253.44-1.64CRP(mg/dL)-5.02ECW/TBW(%)を得ることが可能であった。さらに、終末期がん患者、家族にとっての一つの節目の時期

と考えられ、予後日数中央値（24 日）に近い 4 週間（約 1 カ月）生存における CRP のカットオフ値は 2.0mg/dL、AUC 0.78095、ECW/TBW のカットオフ値は 0.430、AUC 0.72321 であった。

【考察：Discussion】

終末期がん患者の陥る悪液質は、脂肪組織のみならず骨格筋の多大な喪失を呈する[12-13]。さらに、病態生理学的には、経口摂取の減少と代謝異常による負の蛋白・エネルギーバランスを特徴とする”と定義されている[14]。特に、膵がんは、経口摂取量が減少することが多く、容易に悪液質に陥り、全身状態が悪化する[15]。そこで、今回悪液質関連蛋白である血清 TTR、Alb、CRP 値と予後日数との関係を検討した。その結果、終末期膵がん患者に対しては、血清 CRP 値が独立した予後因子であることが判明した。

Shrotriya らは、再発固形腫瘍の予後に関して、CRP が重要な Biomarker であることを、Systematic review で報告している[16]。すなわち、がん組織が増大し、周囲への浸潤、多臓器に転移を起こして、組織の破壊や炎症反応が起こると生体反応として CRP が産生される。血清 CRP 値が高値であるほど、組織破壊や炎症が高いことを示唆している。したがって、血清 CRP 値が高いほど、がん患者の予後が悪くなると報告している。さらに、CRP は、血中 Interleukin(IL)-6 濃度に相関することが知られており、血中 IL-6 濃度は腫瘍増殖活性を反映する[17]。したがって、癌患者における CRP は、腫瘍増殖活性および悪性度を反映すると考えられる。このため、終末期膵がん患者においても、独立した予後因子となったと考えられた。また、CRP は、急性期反応蛋白であり、肺炎などの急性細菌性感染症を併発した場合、急激に血清 CRP 値は、上昇する。幸いなことに、今回の終末期膵がん患者 72 例を詳細に検討した結果、入院時に急性期細菌性感染症を併発している症例は存在しなかった。この観点からも、今回の血清 CRP 値が、終末期膵がん患者の独立した予後因子となった要因の一つであると考えられた。今後、入院時の急性細菌性感染症などを検討する必要が示唆された。

次に、近年サルコペニアの診断基準に、BIA 法が用いられるようになり、体組成分析の重要性が報告されるようになってきている。生体電気インピーダンスは、迅速で、便利な再現性のある測

定技術である。生体の細胞内液と細胞外液の電導率を利用して、異なる周波数の電流を流すことによって、生体組織の抵抗を励起する。人体の様々な部位には様々な電導率があるため、部位別の情報を解析することが可能である。BIA を使用することにより、がん患者の身体組成の動的な変化を追跡及び分析することが可能である。抗がん治療を行っている進行膵がん患者のサルコペニア指標としての BIA の有用性の報告[8]はみられる。今回の、終末期膵がん患者も、悪液質が進行し、サルコペニアは悪化すると考え、検討した。しかし、サルコペニアの指標である SMI と予後との相関関係は認められなかった。これは、浮腫が増強していく終末期がん患者の筋肉組織内にも水分量が増加し、筋肉量の増加として分析されてしまったためではないかと考えられた。

位相角（Phase angle:PhA）は電流が体水分に沿って流れる際に発生する抵抗(レジスタンス)と、細胞膜を通過する際に発生する抵抗(リアクタンス)の位相差である。すなわち、位相角は細胞膜、細胞の構造的な安定さを反映する。位相角に標準値は定められていないが、位相角が低いほど細胞の機能が低下していることを意味する。しかし、位相角は体格と比例し、加齢によって低下していく。今回の終末期膵がん患者に対する検討において、位相角は独立した予後因子ではないことが判明した。これは、がん進行とともに細胞機能は低下し、位相角も低下する可能性が考えられたが、他の因子も加わり、独立した予後因子にはならなかったと考えられた。

ECW/TBW は、体の浮腫の程度を示す優れた指標である。一般に、健康な集団の ECW/TBW は、約 0.38 であり、ECW/TBW が 0.4 を超える人は、水分過剰と定義される[18]。

浮腫を伴う疾患がある場合、主に ECW が増える形でこの数値が高くなる。さらに、加齢・サルコペニアなど栄養状態が悪化した場合は、 ICW が減少するため、ECW/TBW が高くなる。

ECW/TBW は浮腫の指標でありながら、栄養状態や疾患の重症度を示す指標として広く使用されている。Zheng らは、進行がん患者における BIA 法の有用性、特に ECW/TBW≧ 0.40 が予後不良の危険因子であると報告している[19]。しかし、終末期がん患者、特に膵がんのように疾患を特定した予後規定因子に関する体組成分析の有用性を検討した報告は見当たらない。そこで、今回体組

成分析と予後との相関関係を検討した。その結果、ECW/ TBW 値が、終末期膵がん患者の独立した予後因子であることが判明した。これは、終末期膵がん患者は、がんの進行とともに栄養状態が悪化し、ICW が減少することはもちろんのこと、低アルブミン血症による浮腫に伴う ECW の増加を認める。さらに、癌性腹膜炎や胸膜炎により腹水・胸水貯留、多発性肝転移による肝機能障害、心のう液貯留、腎機能障害による浮腫を認め、ECW の増加に繋がってしまう。加えて、悪液質の病態として全身性の炎症反応も高く、血管内皮細胞の脆弱性を招き、血管透過性が亢進することで ECW が増加していることも要因として考えられる。このため、ECW/ TBW 値が、終末期膵がん患者の独立した予後因子となったと考えられた。今後は、入院時簡便に施行できる体組成測定の結果に基づき、体液貯留が認められた症例には、輸液メニュー、利尿薬の投与などの治療介入を試み、効果の是非を検討していきたいと考えている。

最期に、終末期膵がん患者が苛まれる臨床症状と予後につき検討した。Murai らは、悪液質を伴う進行がん患者に対して、general fatigue, anorexia, dyspnea, depression などの臨床症状が予後規定因子であると報告している[4]。Amano らは、がん緩和ケアを受けている患者において、CRP 値上昇とともに、general fatigue, anorexia などの臨床症状も悪化することを報告している[20]。このため、終末期膵がん患者の入院時の臨床症状と予後との関係につき検討した。

終末期膵がん患者においては、depression が多変量解析にて予後と関係する可能性が示唆された。今回の検討は、客観的評価ではなく、終末期膵がん患者に対し、痛みに倣って行った主観的な 10 段階評価を点数化しただけの結果であり、データの曖昧さも危惧され、予後と関係する可能性が示唆されるに留めた。

Murai らは、悪液質を伴う進行がん患者に対して、予後が短い群は、長い群に比較して、Depression をはじめ、General fatigue、Anorexia、Dyspnea、Nausea、Dry mouth などの臨床症状は、有意に高度であると報告している[4]。Depression 状態においては、①家族や医療従事者との癒しの関係の育みがうまくいかない[21]、②生きる希望を失うことが多く、緩和医療においても、消極的な症状コントロールとなってしまう、③無力な態度などが原因となり、

結果として予後との関連性が示唆されたのではないかと考えられた。今後は、入院時評価としての臨床症状（Depression）の結果に基づき、入院早期より多職種の介入、カンファレンスの実施、時には専門医の精神的介入を行い、効果の是非を検討していきたいと考えている。

今回の検討では、①患者の死因が、がんに関連ある慢性的病態か、消化管穿孔、出血、肺炎などの急性病態なのかを詳細に検討していない、②当院入院前入院後の栄養管理が、経口栄養のみか静脈栄養、Total parenteral nutrition などの栄養管理が施行されていたかを検討していない。　このため、終末期膵がん患者の予後日数に影響を及ぼす可能性が考えられ、今後の検討課題と考えられた。

【結論】

終末期膵がん患者において、栄養指標、悪液質指標である血清 TTR、Alb 値は、全例基準値未満の低栄養状態であった。逆に、CRP 値は、多くの症例が、基準値を超える高値の慢性炎症状態であった。多変量解析により、血清 CRP 値、体組成分析 ECW/TBW が独立した予後規定因子であることより、終末期膵がん患者の予後は、栄養状態、サルコペニアよりも慢性炎症や浮腫の程度に関与する可能性が示唆された。さらに、臨床症状では、Depression の程度が予後と関係する可能性があることより、精神的苦痛も考慮しなければいけないと考えられた。

【謝辞：Acknowledgements】統計学的解析に関して、丁寧にご教示いただいた嘉田晃子先生（Associate Prof.）、石原拓磨先生（Assistant Teacher)、松田勇紀先生（Assistant Prof.）に心より感謝申し上げます。

【利益相反】著者は、本研究に関わる企業や営利目的とした団体との利益相反はありません。

【Reference】

1. Cancer statics in Japan　2024　Foundation for Promotion of Cancer Research　https://www.fpcr.or.jp/
2. Dewys WD, Begg C, Lavin PT, et al. Prognostic effect of weight loss prior to chemotherapy in cancer patients. Eastern Cooperative Oncology Group. Am J Med 1980; 69: 491-7.
3. Mitsunaga S, Ikeda M, Shimizu S, Ohno I, Takahashi H, Okuyama H, Ueno H, Morizane C, Kondo S, Sakamoto Y, Okusaka T, Ochiai A. C-reactive protein level is an indicator of the aggressiveness of advanced pancreatic cancer. Pancreas 2016; 45: 110-6.
4. Murai M, Higashiguchi T, Futamura A, Ohara H, Tsuzuki N, Itani Y, Kaneko T, Chihara T, Shimpo K, Nakayama N.　Interleukin-8 and clinical symptoms can be prognostic indicators for advanced cancer patients with cachexia. Fujita Med J 2020; 6: 117-21.
5. McMillan DC. The systemic inflammation-based Glasgow Prognostic Score: a decade of experience in patients with cancer. Cancer treat Rev 2013; 39: 534-40.
6. Miura T, Matsumoto Y, Hama T, et al. Glasgow prognostic score predicts prognosis for cancer patients in palliative settings: a subanalysis of the Japan-prognostic assessment tools validation (J-ProVal) study. Support Care Cancer 2015; 23: 3149-56.
7. Miura T, Amano K, Shirado A, Baba M, Ozawa T, Nakajima N, Suga A, Matsumoto Y, Shimizu M, Shimoyama S, Kuriyama T, Matsuda Y, Iwashita T, Mori I, Kinoshita H.　Low Transthyretin Levels Predict Poor Prognosis in Cancer Patients in Palliative Care Settings. Nutr Cancer 2018; 70: 1283-9.
8. Tozuka Y, Ueno M, Kobayashi S, Morimoto M, Fukushima T, Sano Y, Kawano K, Hanaoka A, Tezuka S, Asama H, Moriya S, Morinaga S, Ohkawa S, Maeda S. Prognostic significance of sarcopenia as determined by bioelectrical impedance analysis in patients with advanced pancreatic cancer receiving gemcitabine plus nab-paclitaxel: A retrospective study. Oncol Lett 2022;24:375.
9. Chen LK, Woo J, Assantachai P, et al. Asian Working Group for

Sarcopenia: 2019 Consensus Update on Sarcopenia Diagnosis and Treatment. J Am Med Dir Assoc 2020;21: 300-7.

10. Caraceni A, Cherny N, Fainsinger R, Kaasa S, Poulain P, Radbruch L, De Conno F. Pain measurement tools and methods in clinical research in palliative care: recommendations of an Expert Working Group of the European Association of Palliative Care. J Pain Symptom Manage 2002; 23: 239–55.
11. Bruera E, Macdonald S. Audit methods: the Edmonton symptom assessment. In: Higginson I. Clinical Audit in Palliative Care. Oxford: Radcliffe Medical; 1993: 61–77.
12. Tisdale MJ. Pathogenesis of cancer cachexia. J Support Oncol 2003; 1: 159-68.
13. Evans WJ, Morley JE, Argilés J, et al. Cachexia: a new definition. Clin Nutr 2008; 27: 793-9.
14. Radbruch L, European Palliative Care Research Collaborative. Clinical practice guidelines on cancer cachexia in advanced cancer patients with a refractory cachexia. Aachen: Department of Palliative Medicinen/ European Palliative Care Research Collaborative; 2010.
15. Baracos V, Martin L, Korc M, Guttridge DC, Fearon KCH. Cancer-associated cachexia. Nat Rev Dis Primers 2018; 4: 17105.
16. Shrotriya S, Walsh D, Bennani-Baiti N, Thomas S, Lorton C. C-Reactive Protein is an important biomarker for prognosis tumor recurrence and treatment response in adult solid tumors: A Systematic Review. PLoS One 2015; 10: e0143080.
17. Kinoshita T, Ito H, Miki C. Serum interleukin-6 level reflects the tumor proliferative activity in patients with colorectal carcinoma. Cancer 1999; 85: 2526-31.
18. Kyle UG, Bosaeus I, De Lorenzo AD, Deurenberg P, Elia M, Gómez JM, Heitmann BL, Kent-Smith L, Melchior JC, Pirlich M, Scharfetter H, Schols AM, Pichard C. Bioelectrical impedance analysis--part I: review of principles and methods. Clin Nutr 2004; 23:1226-43.
19. Zheng K, Lu J, Liu X, Ji W, Liu P, Cui J, Li W. The clinical

application value of the extracellular-water-to-total-body-water ratio obtained by bioelectrical impedance analysis in people with advanced cancer. Nutrition 2022; 96: 111567.

20. Amano K, Maeda I, Morita T, et al. C-reactive protein, symptoms and activity of daily living in patients with advanced cancer receiving palliative care. J Cachexia Sarcopenia Muscle 2017; 8: 457–65.

21. Pinquart1 M, Duberstein PR. Depression and cancer mortality: a meta-analysis. Psychol Med 2010; 40: 1797–810.

legend for figures

Figure 1 Overall assessment score

Figure.2-A Comparison of survival rate divided by median CRP level

Figure.2-B Comparison of survival rate divided by median ECW/TBW level

Figure.3 Setting cut-off value using ROC curve (4 weeks survival)

Table 1. Characteristics of patients

Table. 2-A Significant difference between laboratory data and prognosis at admission

Table. ２-B Significant difference between Body composition analysis and prognosis at admission

Table. 3 Correlation between covariates –Spearman rank correlation coefficient

Table. 4-A Multivariable Cox regression analyses for mortality in terminal pancreatic cancer patients

Table. 4-B Multivariable Cox regression analyses for mortality in terminal pancreatic cancer patients

Table. 5 Significant difference between Clinical symptoms and prognosis at admission

Table. 6 Multivariable Cox regression analyses for mortality in terminal pancreatic cancer patients
